# SELF-REPORTED WELL-BEING AMONG OLDER ADULTS WITH A PERSONAL OR FAMILY HISTORY OF INCARCERATION

**DOI:** 10.1093/geroni/igae098.3217

**Published:** 2024-12-31

**Authors:** Louisa Holaday, Kimberly Stone, Brita Roy, Brie Williams, Albert Siu, Emily Wang

**Affiliations:** Icahn School of Medicine at Mount Sinai, New York, New York, United States; Icahn School of Medicine at Mount Sinai, New York, New York, United States; NYU Langone Health, New York, New York, United States; University of California San Francisco, San Francisco, California, United States; Icahn School of Medicine at Mount Sinai, New York, New York, United States; Yale University, New Haven, Connecticut, United States

## Abstract

The United States has one of the highest incarceration rates in the world. Prior work has demonstrated adverse health effects associated with personal or family history of incarceration, but none has focused on older adults. We conducted a cross-sectional study using data from the nationally representative Family History of Incarceration survey. Among the 1,114 adults aged 55 or older, we tested the association between personal incarceration and/or family member incarceration and overall; financial; social; physical; and mental wellbeing controlling for age; gender; race/ethnicity; income; education; employment; and marital status. We accounted for time since last incarceration, and duration of incarceration. Overall, 21% of respondents had been incarcerated (of whom 78% returned to the community over 10 years ago), and 64% had had an immediate family member incarcerated (of whom 30% were incarcerated 1 year or longer). “Thriving” overall wellbeing, which is associated with life expectancy, was less common among those with personal or family incarceration exposure (63.3% of those without incarceration exposure vs. 47.7-55.7% of those with incarceration exposure). In fully adjusted models, personal or family incarceration exposure was associated with lower odds of reporting “excellent” or “very good” physical health (OR: 0.50-0.55; all P< 0.05); and “thriving” social wellbeing (OR: 0.44-0.60; all P< 0.008). There existed a dose response relationship between longer durations of family member incarceration and lower wellbeing. Exposure to incarceration is independently associated with self-reported physical health and social wellbeing among older adults. Policy makers should consider methods to mitigate the harms associated with incarceration.

